# Common MicroRNA Signatures in Cardiac Hypertrophic and Atrophic Remodeling Induced by Changes in Hemodynamic Load

**DOI:** 10.1371/journal.pone.0014263

**Published:** 2010-12-09

**Authors:** Ali El-Armouche, Alexander Peter Schwoerer, Christiane Neuber, Julius Emmons, Daniel Biermann, Thomas Christalla, Adam Grundhoff, Thomas Eschenhagen, Wolfram Hubertus Zimmermann, Heimo Ehmke

**Affiliations:** 1 Department of Pharmacology, University Medical Center Goettingen (UMG), Goettingen, Germany; 2 Department of Experimental and Clinical Pharmacology and Toxicology, University Medical Center Hamburg-Eppendorf, Hamburg, Germany; 3 Department of Vegetative Physiology and Pathophysiology, University Medical Center Hamburg-Eppendorf, Hamburg, Germany; 4 Heinrich-Pette-Institute for Experimental Virology and Immunology, Hamburg, Germany; Victor Chang Cardiac Research Institute, Australia

## Abstract

**Background:**

Mechanical overload leads to cardiac hypertrophy and mechanical unloading to cardiac atrophy. Both conditions produce similar transcriptional changes including a re-expression of fetal genes, despite obvious differences in phenotype. MicroRNAs (miRNAs) are discussed as superordinate regulators of global gene networks acting mainly at the translational level. Here, we hypothesized that defined sets of miRNAs may determine the direction of cardiomyocyte plasticity responses.

**Methodology/Principal Findings:**

We employed ascending aortic stenosis (AS) and heterotopic heart transplantation (HTX) in syngenic Lewis rats to induce mechanical overloading and unloading, respectively. Heart weight was 26±3% higher in AS (n = 7) and 33±2% lower in HTX (n = 7) as compared to sham-operated (n = 6) and healthy controls (n = 7). Small RNAs were enriched from the left ventricles and subjected to quantitative stem-loop specific RT-PCR targeting a panel of 351 miRNAs. In total, 153 miRNAs could be unambiguously detected. Out of 72 miRNAs previously implicated in the cardiovascular system, 40 miRNAs were regulated in AS and/or HTX. Overall, HTX displayed a slightly broader activation pattern for moderately regulated miRNAs. Surprisingly, however, the regulation of individual miRNA expression was strikingly similar in direction and amplitude in AS and HTX with no miRNA being regulated in opposite direction. In contrast, fetal hearts from Lewis rats at embryonic day 18 exhibited an entirely different miRNA expression pattern.

**Conclusions:**

Taken together, our findings demonstrate that opposite changes in cardiac workload induce a common miRNA expression pattern which is markedly different from the fetal miRNA expression pattern. The direction of postnatal adaptive cardiac growth does, therefore, not appear to be determined at the level of single miRNAs or a specific set of miRNAs. Moreover, miRNAs themselves are not reprogrammed to a fetal program in response to changes in hemodynamic load.

## Introduction

Physiological and pathological changes in cardiac workload can cause prominent alterations in gene expression [1]. These adaptive genetic responses have been well described for cardiac hypertrophy and are characterized by an elevation of abundance of fetal genes, for example in β-myosin heavy chain (β-MHC) [2]. Remarkably, opposite changes in cardiac workload (i.e. hemodynamic overloading versus unloading), although associated with distinct phenotypes (hypertrophy versus atrophy), produce strikingly similar transcriptional changes [3]. This principle finding was confirmed by several groups demonstrating uni-directional changes in hypertrophy-associated mRNAs - including ANP, β-MHC and α-skeletal actin - in hypertrophied and atrophied hearts (for review see [4]). These observations suggested that respective changes, although characteristic for the remodeling process, may have a limited role in regulating the direction of cardiac plasticity.

MicroRNAs (miRNAs) have recently been identified as superordinate regulators of global gene networks acting mainly at the translational level. These small, endogenous, non-coding RNA molecules are capable of suppressing gene expression in a sequence-specific manner. Depending on the grade of complementarity with the target mRNA, miRNAs either repress translation or induce degradation of mRNA. Usually the interaction of a miRNA with its target mRNA is characterized by mismatches, which then leads to translation repression rather than a decrease in mRNA amount [5]. Recent studies have documented an essential role for various miRNAs in modulating key components of the hypertrophic process in the heart [6]–[13]. Moreover, miRNA expression profiling in pathologically hypertrophied or failing hearts in humans and mice suggests that miRNA expression changes may be typical for specific cardiac diseases [11], [14]–[19]. Similar studies on hemodynamically unloaded, atrophied hearts, however, do not exist yet. Heterotopic heart transplantation has been established previously as a reliable methodology to induce atrophic growth *in vivo* [2], [3], [20]–[22].

Given that miRNAs can regulate global gene networks at the translational level, we hypothesized that the switch towards either a hypertrophic or atrophic phenotype is associated with the activity of specific sets of miRNAs. To test this hypothesis we analyzed the expression of a broad range of miRNAs using quantitative stem-loop RT-PCR arrays on left ventricular samples from rat hearts, in which hypertrophy (mechanical overload) or atrophy (mechanical unloading) of comparable extent were induced. To evaluate the degree of overlap between the miRNA expression pattern(s) activated during postnatal cardiac remodeling with those active during fetal development we also investigated the expression of miRNAs in rat hearts at embryonic day 18.

## Results

### Highly concordant miRNA expression in hypertrophic and atrophic hearts

**Figure 1:**
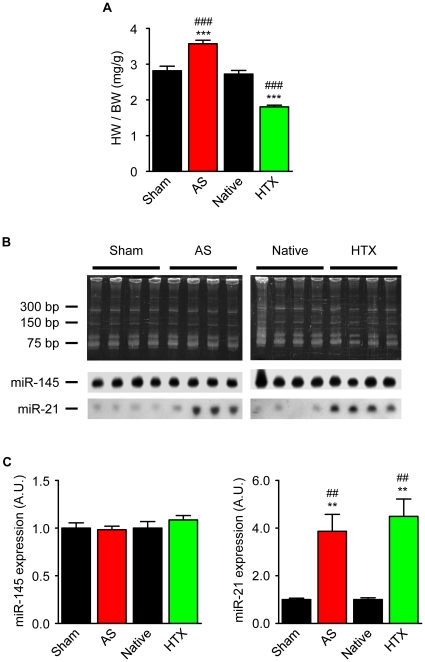
Effect of altered cardiac workload on heart weight and the expression of miR-145 and miR-21. (A) Relative heart weights (heart weight to body weight ratio, HW/BW) of animals undergoing ascending aortic stenosis (AS, n = 7) and heterotopic heart transplantation (HTX, n = 7). *** P<0.001 versus Native (n = 7), ### P<0.001 versus Sham (n = 6), one-way ANOVA, followed by a Bonferroni *posthoc* test. (B) Representative Northern blot and (C) densitometric analysis of miR-145 and miR-21 expression in the different groups. ** P<0.01 versus Native; ## P<0.01 versus Sham, one-way ANOVA followed by Bonferroni *posthoc* test.

To assess the hemodynamic load-induced changes on miRNA pattern, male Lewis rats were subjected to increased cardiac workload by ascending aortic stenosis (AS) or to decreased cardiac workload by heterotopic heart transplantation (HTX). Hearts from sham-operated animals (Sham) served as controls for AS. Not manipulated orthotope hearts of the HTX recipients (Native) served as controls for HTX. As expected, AS (n = 7) and HTX (n = 7) resulted in a higher (+26±3%) and a lower (-33±2%) relative heart weight, respectively (Figure 1A). The relative heart weights of the control groups (Native: 2.7±0.1 mg/g, n = 7; Sham: 2.8±0.1 mg/g, n = 6) did not differ.

To identify miRNAs that are differentially expressed in hypertrophic and atrophic hearts, we performed specific stem-loop RT-PCR analysis targeting a panel of 351 different mature miRNAs (Table S1) in left ventricular tissue. Out of all miRNAs investigated, 153 miRNAs could be unambiguously detected; a mean cycle of threshold (CT) value of 35 cycles was defined as a cut-off for reliable miRNA detection (CT value defines the threshold, i.e. the cycle number when exponential PCR product amplification occurs; Table S1).

To focus the following expression analysis on miRNAs that have recently been shown to be expressed in the human or rodent cardiovascular system [11], [12], [14], [15], [23], a reference list was compiled containing 81 miRNAs. The miRNA array used in this study was based on human mature miRNA sequences (see Table S1). To exclude that this species difference impairs the expression analysis, a sequence comparison was performed using the current version (release 16) of the Sanger miRBase (see Table S2). According to this database, 72 miRNAs are 100% conserved between these species. Thus, independent of the species, the primers used by the human miRNA array fully detect these miRNAs. Three miRNAs of this initial cardiovascular reference list, however, are not 100% conserved between human and rat (miR-1, miR-211 and miR-424) and six miRNAs have not yet been registered for rats (miR-7g, miR-149, miR-15a, miR-199b, miR-372 and miR-486). Nonetheless, stable expression could be detected for these nine miRNAs in all experimental groups, indicating that (1) the single nucleotide polymorphisms did not abrogate the detection efficiency and (2) the yet unannotated miRNAs have homologues in rat. To fully exclude the possibility of incorrect expression analysis and subsequent interpretation, however, these miRNAs were excluded. Table 1 lists the remaining 72 miRNAs which met the above described inclusion criteria and were, therefore, included in the following data analysis.

**Table 1:** miRNA reference list.

let-7a	let-7b	let-7c	let-7d	let-7e	let-7f	miR-100
miR-101	miR-103	miR-107	miR-10a	miR-10b	miR-125b	miR-126
miR-130a	miR-133a	miR-133b	miR-139	miR-140	miR-143	miR-146a
miR-15b	miR-16	miR-17-3p	miR-17-5p	miR-181b	miR-182	miR-191
miR-195	miR-199a	miR-19a	miR-19b	miR-208	miR-20a	miR-21
miR-210	miR-214	miR-218	miR-22	miR-221	miR-222	miR-224
miR-23a	miR-23b	miR-24	miR-25	miR-26a	miR-26b	miR-27a
miR-27b	miR-28	miR-29a	miR-29c	miR-30a-3p	miR-30a-5p	miR-30b
miR-30c	miR-30d	miR-30e-3p	miR-30e-5p	miR-31	miR-320	miR-342
miR-378	miR-422b	miR-423	miR-451	miR-484	miR-497	miR-92
miR-93	miR-99b					

List of miRNAs that have been detected with an average CT value ≤35 cycles in all experimental groups and that have been shown (or are discussed) to have a potential impact on the cardiovascular system (see Results). The analysis of miRNA expression has been restricted to these 72 miRNAs which are fully conserved between humans and rats (Table S2).

The relative expression of each of these miRNAs was then normalized to miR-145, which was found to display a robust and stable expression within all experimental groups (CT ∼26, see Table S1). The internal control miR-145 and the hitherto best characterized miRNA relevant for the cardiovascular system, miR-21, were analyzed by Northern blotting (Figure 1B and 1C), which confirmed the data of the quantitative reverse transcriptase-polymerase chain reaction (qRT-PCR) and previous publications [11], [18], [24].

**Figure 2:**
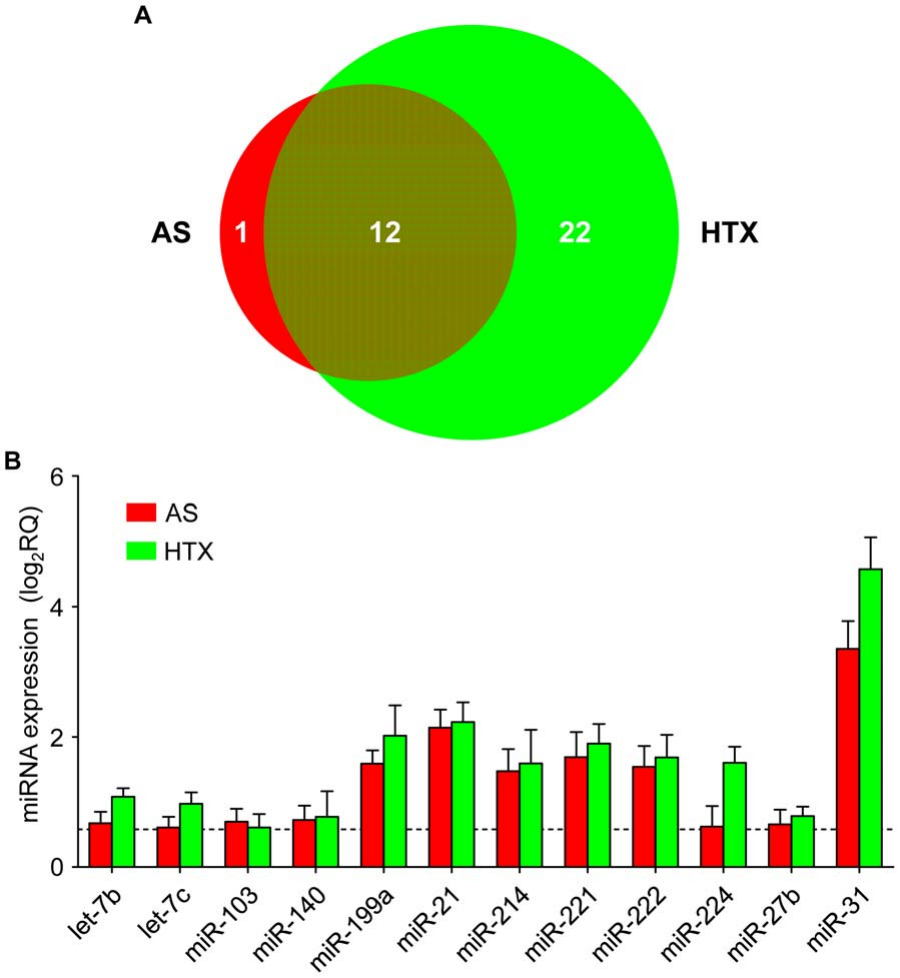
MiRNA expression in mechanical load-induced cardiac hypertrophy and cardiac atrophy. (A) Venn diagram of up-regulated miRNAs in hypertrophied (ascending aortic stenosis, AS) and atrophic (heterotopic heart transplantation, HTX) hearts. For the complete list of regulated miRNAs see Table S4. (B) Relative expression data of all miRNAs that displayed a≥50% up-regulation in both intervention groups (AS and HTX).

MiRNA abundance in the two control groups (Native and Sham) did not differ in any of the potentially relevant miRNAs (two-way ANOVA, followed by Bonferroni *posthoc* test). Figure S1 illustrates the ΔCT values (CT_sample_-CT_miR-145_). This plot reveals a strict linear relationship of the quantitative miRNA expression between both groups with a slope of 1.00±0.01 (r^2^ = 0.99, n = 72). Therefore, both groups were considered identical and were pooled to form a single control group (Control). The miRNA expression data of the two intervention groups (AS, HTX) were then normalized to the Control expression data (log_2_RQ, Table S3). Based on the number of tissue samples and the resolution of the quantification method, we defined a difference in miRNA abundance by ±50% from Control, corresponding to ±0.58 on the log_2_RQ scale, as up- and down-regulation, respectively. Accordingly, a total of 40 miRNAs were regulated in hypertrophied (AS) and atrophied (HTX) hearts, respectively (Table S4). The vast majority of these miRNAs (n = 35) exhibited increased expression levels (Figure 2A), comprising 13 miRNAs in hypertrophied hearts (AS) and 34 miRNAs in atrophied hearts (HTX). 12 of these miRNAs were ≥50% up-regulated in both groups with particularly strong increases in expression for miR-199a, miR-21, miR-214, miR-221, miR-222, and miR-31 (Figure 2B). Moreover, 22 miRNAs were ≥50% up-regulated selectively in HTX hearts and one miRNA in AS hearts. By contrast, the relative changes of the five down-regulated miRNAs were modest, barely exceeding a log_2_RQ value of -1, and were restricted to the AS group (Figure S2).

**Figure 3:**
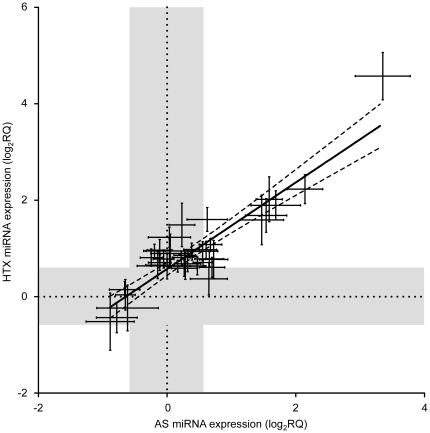
Highly overall concordance of miRNA expression in hypertrophic and atrophic hearts. Depicted are relative expression data (log_2_RQ) of ≥50% up- or down-regulated miRNAs detected in hearts from HTX versus AS animals (see Table S4). The fit curves represent a linear regression±95% confidence intervals (r^2^ = 0.79, slope = 0.9±0.07; n = 40). The gray areas denote relative expression values ranging from -0.58 to 0.58 log_2_RQ.

The Venn-analysis only refers to qualitative changes in the expression. However, since quantitative effects may be biologically more important, we plotted absolute changes in the expression levels of all individual miRNAs measured in atrophied hearts versus those measured in hypertrophied hearts. Surprisingly, this analysis revealed a highly concordant regulation of individual miRNAs in AS and HTX (r^2^ = 0.79, slope = 0.9±0.07; n = 40, Figure 3). Notably, no single miRNA was found to be regulated in opposite directions in response to AS and HTX, respectively.

To identify potential targets of (1) the miRNAs that were commonly activated by cardiac atrophy and hypertrophy and of (2) the miRNAs activated only during cardiac atrophy, downstream targets were predicted using the TargetScan algorithm which is based on human miRNA targets [25]–[27]. By calculating the most likely mRNA targets, the prediction algorithm ranked ∼3000 different genes potentially targeted by the 12 miRNAs up-regulated in both remodeling processes (see Table S5) and ∼5600 different genes targeted by the 22 miRNAs up-regulated only in atrophic hearts (see Table S6). Analysis of related gene ontology terms did not reveal a significant overrepresentation of specific functional categories (data not shown).

### Hypertrophic and atrophic remodeling differ markedly from the fetal miRNA expression pattern

**Figure 4:**
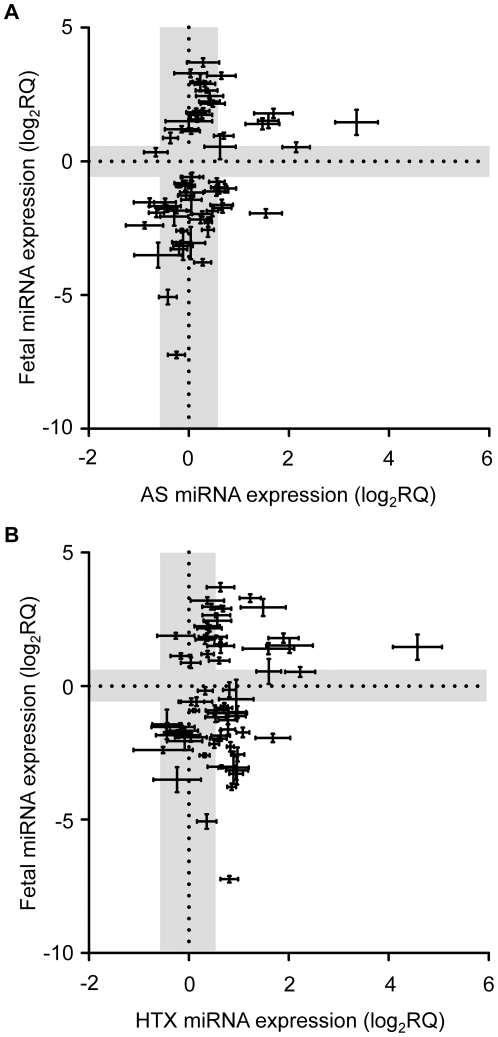
Hypertrophic and atrophic miRNA remodeling differs markedly from the developmental fetal miRNA expression pattern. (A) Relative expression of miRNAs in AS versus Fetal hearts. (B) Relative expression pattern of miRNAs in HTX versus Fetal hearts. The gray areas denote relative expression values ranging from −0.58 to 0.58 log_2_RQ. Only data of ≥50% up- or down-regulated miRNAs (see Table S7) are plotted.

Several studies have reported that both cardiac unloading and overloading induce changes in gene expression that mimic the fetal gene program in important aspects, e.g. genes involved in cardiac metabolism or in contractile properties [3], [4]. To test the hypothesis that the common miRNA signature induced by altered cardiac workload represents a reprogramming towards a fetal gene response we analyzed the miRNA expression in fetal hearts from male Lewis embryos at embryonic day 18 (Fetal, ED18, n = 6). All data were normalized to miR-145 and compared with expression levels observed in the Control hearts (Table S3). Notably, the majority of miRNAs (61 out of 72) differed between Fetal and adult hearts (Table S7). Similarly, the concordance of miRNA levels in Fetal hearts and in both intervention groups was surprisingly low (Fetal versus AS - Figure 4A; Fetal versus HTX – Figure 4B). Indeed, neither of the data sets could be described by any linear or non-linear relationship, indicating that neither AS nor HTX merely induces a gross re-expression of a fetal miRNA program.

## Discussion

The adult mammalian heart can considerably increase (hypertrophy) or decrease (atrophy) its size in response to alterations in mechanical workload. In this study we analyzed miRNA abundance in left ventricular myocardium using quantitative stem-loop RT-PCR based miRNA expression arrays in two well established *in vivo* models of altered cardiac workload, i.e. AS (model of cardiac hypertrophy [3], [28]–[30]) and HTX (model of cardiac atrophy [2], [3], [20]–[22]). We discovered that opposite changes in cardiac workload associated with nearly maximum changes in left ventricular mass (+26% versus -33%) induce highly concordant miRNA expression profiles without any miRNAs being regulated in opposite directions.

One may argue that the miRNA expression pattern associated with cardiac hypertrophy does not necessarily need to be the exact inverse of that associated with cardiac atrophy. However, not only the qualitative (up- or down-regulation) but also the quantitative changes in miRNA expression were highly concordant in the hypertrophied and atrophied hearts. The relation of the relative gene expression levels in hypertrophied and atrophied hearts could be well described by a linear function with a slope of close to one (see Figure 3). These findings suggest that any change in cardiac workload - independent of its direction - induces a common miRNA remodeling program. Similar common responses to changes in hemodynamic load have also been described for the so-called fetal genes (e.g. ANP, β-MHC, and α-skeletal-actin [3]) as well as for cellular remodeling processes (e.g. in cardiac electrophysiology [21]).

Such a concept, however, would also imply that the direction of growth plasticity in postnatal cardiomyocytes is not determined at the level of miRNA transcription. It must be emphasized here that miRNA expression profiling studies cannot define possible functional distinctions between constant "pools" of miRNAs, *de novo* synthesized miRNAs, and posttranscriptional miRNA processing [12]. Thus, an apparent discordance between qRT-PCR findings and plasticity changes could reflect differences in miRNA localization, processing and/or stability, which may be more critical in determining the overall direction of adaptive cardiomyocyte plasticity in the adult heart [2].

The cellular remodelling induced by AS and HTX may be associated with increased fibrosis and autophagy which could explain some of the similarity in the gene responses to both interventions. MiR-29, miR-133 and miR-30c are the most strongly fibrosis-associated miRNAs targeting a number of extracellular-matrix-related mRNAs [31], [32]. These miRNAs, however, were not regulated under our experimental conditions arguing against a pathological activation of fibroblasts. Autophagy has been shown to be activated in cardiac remodeling in virtually every form of myocardial disease and appears to serve as a protective mechanism [33]–[35]. However, functional regulation of autophagy by specific miRNAs in cardiac myocytes has not been determined yet.

Previous reports on miRNA expression in the cardiovascular system focused on pathological cardiac hypertrophy and failing hearts [14]–[16]. Biomechanical stress and neurohumoral factors are considered to be the two most important triggers of cardiac hypertrophy, with neurohumoral activation primarily involved in pathological cardiac growth [36]. Ascending aortic stenosis [28], [29] and heterotopic heart transplantation induce large functional changes in hemodynamic load without major neurohumoral activation. Accordingly, the alterations in miRNA expression patterns observed in the present study most likely represent the physiological adaptive responses to altered cardiac workload. However, differences may exist between this miRNA response pattern and that induced in hearts undergoing a pathological structural remodeling.

Our data on specific miRNA changes induced by AS in rats are in very good accordance with other studies on mouse hearts subjected to transverse aortic banding/constriction. Several miRNAs, including miR-21, miR-27, miR-31, miR-199a, miR-214 and miR-222 were up-regulated in these mouse models in the same directions and to similar extents as observed in this study [11], [18]. This indicates that species and model differences do not have a major impact on miRNA regulation.

Interestingly, we observed that the total number of miRNAs being ≥50% up- or down-regulated as compared to control differed between HTX and AS. Overall, HTX hearts displayed a broader activation pattern of moderately regulated miRNAs (Figure 2A and Figure S2). One possible reason for this difference in global miRNA regulation may be that the degree of atrophic versus hypertrophic remodeling does not directly match with the absolute changes in left ventricular mass.

Cardiac overloading and unloading have both been shown to reactivate the expression of fetal genes [3], [4]. In the present study, however, neither condition produced a miRNA expression pattern which resembled the fetal miRNA expression pattern (see Figure 4). This finding suggests that miRNAs themselves - in contrast to mRNAs - are not reprogrammed to a fetal phenotype in response to changes in biomechanical stress. This does not support the general idea that fetal gene reprogramming induced by workload dependent cardiac stress is a global event involving all molecular and transcriptional regulatory levels of the heart. In contrast to our study that focused on miRNA regulation as a response to short term cardiac overload without secondary, adverse effects from heart failure, chronic cardiac diseases may indeed induce a fetal miRNA expression pattern. A recent study by Thum et al. analyzing miRNA levels in failing and in fetal human hearts reported that >80% out of ∼350 miRNAs showed concordant differences compared to healthy controls [15].

Some limitations of this study should be mentioned. First, the expression of miRNAs was analyzed using primers based on the human mature miRNA sequences. Only miRNAs that have a homologue in rats and that are 100% conserved between humans and rats were included in this study (Table S2). Thus, while these criteria led to the exclusion of nine miRNAs (see Results), the expression data of the 72 miRNAs remaining in this study (Table 1), are not affected by the use of human based miRNA arrays. However, it cannot be excluded that single miRNAs relevant for the development of hypertrophy and/or atrophy specifically in rats may have been overlooked. This limitation is caused by the current, rather poor, annotation rate for miRNAs in the miRBAse and, thus, affects all miRNA expression analysis carried out in rats. Second, the duration of overloading (7 days) and unloading (14 days) - although causing comparable degrees of changes in left ventricular mass (±∼30%) - may not correspond to the time at which changes in miRNA expression have their major impact. It has been shown, however, that several contractile and metabolic transcripts remain up- or down-regulated at remarkably constant levels between day 7 and day 28 in overloaded and unloaded hearts [3] which suggests that the regulation of the transcriptome has attained a steady state during this time span after a sudden change in hemodynamic load. Third, the target prediction analysis did not reveal any specific sets of genes involved in defined cell-biological aspects. This is very likely due to the complex nature of cardiac remodeling and due to the fact that the existing computational algorithms are still challenging [37]. It should, however, be pointed out that the predictions were carried out for the evolutionary conserved miRNAs. Thus, although based on the known human miRNA targets, the target prediction algorithm should reveal all functionally important targets which are fully conserved across species. Finally, this study does not exclude that atrophic and hypertrophic remodeling may be controlled by a fine-tuning within a large network involving minimal changes (below ±50%) in a large number of individual miRNAs.

In conclusion, this study provides unexpected evidence that in adult hearts changes in biomechanical stress *per se* induce a specific miRNA remodeling pattern which, however, does not determine the direction of the growth response in postnatal cardiomyocytes and does not resemble the fetal miRNA program.

## Materials and Methods

### Animal models and tissue samples

All experiments were performed in syngenic male Lewis rats (Charles River, Germany). Cardiac hypertrophy was induced in adult rats (224±6 g, n = 7) by ascending aortic stenosis (AS) for 7 days as previously described [30]. Hearts from sham-operated animals (Sham; 242±8 g, n = 6) were also excised after 7 days. Cardiac atrophy was induced in adult rats (donor rats: 236±3 g, n = 7; recipient rats: 236±4 g, n = 7) by heterotopic heart transplantation (HTX) as previously described [2], [3], [20]–[22]. HTX hearts and native *in situ* hearts (Native, n = 7) from the recipient animals were explanted after 14 days of unloading. Ventricular tissue of male fetal hearts was harvested at embryonic day 18 from pregnant Lewis rats (Fetal, n = 6). All animal experiments were approved by local authorities (Ministry for Social Affairs, Family, Health and Consumer Protection, Hamburg, Germany, approval number: 02/04).

### Gene expression analysis

Expression levels of miRNAs were determined by quantitative reverse transcriptase-polymerase chain reaction (qRT-PCR) [38]. Small RNAs from left ventricular tissue were enriched by using *mir*Vana™ miRNA Isolation Kit (Ambion, Inc.). qRT-PCR was performed with cDNA generated from 100 ng of total RNA by using Multiplex RT for TaqMan MicroRNA Assays (Applied Biosystems) with highly-specific stem-loop reverse-transcriptase (RT) primers for 351 different mature human miRNAs (see Table S1 for the context sequences). Table S2 shows that all miRNAs which have previously been shown to be expressed in cardiovascular tissues (n = 72, see also Table 1) are fully conserved between human and rat (release 16 of the Sanger miRBase). Thus, the relevant miRNAs are fully detected by the human based primers irrespective of the species. Real-time PCR reactions were executed on each target using TaqMan Low Density Array (Applied Biosystems). The 7900HT Fast Real-Time PCR System and SDS software were used to amplify and detect specific products. Northern blot analysis was performed as previously described [39].

### Data analysis and normalization

CT values of miRNA expression obtained from qRT-PCR analysis were processed using the SDS software 2.3 (Applied Biosystems, Foster City, CA), Excel (Microsoft, Redmond, WA) and PRISM 5 (GraphPad, San Diego, CA). Only miRNAs with mean CT values ≤35 in all groups were included in this study (n = 153, Table S1). All CT values were normalised to miR-145 by the equation: 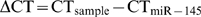



The relative expression (RQ) of each miRNA in the target groups (AS, HTX, Fetal) was calculated by the equation: 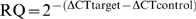



The Control group consisted of the pooled data from Sham and Native hearts (see Results). For means of clarity, the relative expression data are given as log_2_RQ within the manuscript (unless stated otherwise, Table S3).

### MiRNA target prediction

MiRNA targets were predicted using the current version (release 5.1) of the TargetScan algorithm (http://www.targetscan.org) as described previously [26], [27] (see [25] for a review). Briefly, the algorithm calculates a "context score" for each predicted miRNA hit, which corresponds to the log_2_ ratio (hence lower values mean stronger repression) of expected regulation in experimental systems [26]. A cumulative (or "total") context score for a given transcript can thus be calculated by summation of the score values of individual sites. For the prediction of jointly regulated transcripts, predictions were made first for individual miRNA seed families, and a joint or "cumulative" context score was then calculated by summation of the "total" context scores of individual seed family hits.

### Statistical analysis

Data are given as mean±SEM. Unless stated otherwise, statistical significance was calculated by one-way ANOVA followed by Bonferroni *posthoc* test for multiple comparisons using PRISM 5 (GraphPad, San Diego, CA).

## Supporting Information

Table S1 Expression (CT) of all miRNAs included in the array (n = 351). *n* indicates the number of valid samples.
(0.15 MB XLS)

Table S2 Comparison between the mature human miRNA sequences (as used in the array) and the mature rat miRNA sequences. Sequences have been retrieved from the current version (release 16) of the Sanger miRBase (last access: 30.10.2010). None marks Sequences without annotated rat homologues. The number of nucleotide mismatches is presented in the last column. miRNAs with 0 mismatches are 100% conserved between humans and rats.
(0.04 MB XLS)

Table S3 Relative expression (log2RQ) of all miRNAs with implications for the cardiovascular system (n = 82, Table 1). The expression was normalized to the housekeeping miR-145 and to the expression in the Control group (see Results and Materials and Methods sections of the manuscript). n indicates the number of samples.
(0.06 MB XLS)

Table S4 MiRNAs that were ≥50% up- or down-regulated in AS and/or HTX hearts.
(0.03 MB XLS)

Table S5 Potential mRNA targets of the 12 miRNAs up-regulated in AS and HTX hearts (see also Table S4). MiRNA target transcripts were predicted using the TargetScan algorithm as described in Material & Methods. For each individual transcript and miRNA seed family, the rightward columns of the table list the number of evolutionary conserved and non-conserved hits (further classified according to the type of seed match, i.e. 8mer, 7mer-m8 or 7mer-a1), the total context score of the prediction (a measure of the predicted efficacy of targeting) as well as the aggregate PCT (preferentially conserved targeting) value, which is a metric that considers the phylogenetic relationship between species in which the site is conserved. The leftmost columns titled ‘All miRNAs’ give the total number and names of miRNAs for which target sites were predicted in a particular transcript, as well as the sum counts of conserved and non-conserved hits (note that the table only lists transcripts for which at least one evolutionary conserved target site was predicted). The predicted targets are sorted according to the "Cumulative Context Score" column, which corresponds to the sum of the individual context score values and thus is a simulated measure of combined targeting by all upregulated miRNAs. The column "Min. Aggregate PCT" indicates the minimal PCT value achieved by any of the miRNAs predicted to target a given transcript.
(4.97 MB XLS)

Table S6 Potential mRNA targets of the 22 miRNAs up-regulated in hearts of the HTX but not the AS group (see also Table S4). MiRNA target transcripts were predicted using the TargetScan algorithm as described in Material & Methods. For each individual transcript and miRNA seed family, the rightward columns of the table list the number of evolutionary conserved and non-conserved hits (further classified according to the type of seed match, i.e. 8mer, 7mer-m8 or 7mer-a1), the total context score of the prediction (a measure of the predicted efficacy of targeting) as well as the aggregate PCT (preferentially conserved targeting) value, which is a metric that considers the phylogenetic relationship between species in which the site is conserved. The leftmost columns titled ‘All miRNAs’ give the total number and names of miRNAs for which target sites were predicted in a particular transcript, as well as the sum counts of conserved and non-conserved hits (note that the table only lists transcripts for which at least one evolutionary conserved target site was predicted). The predicted targets are sorted according to the "Cumulative Context Score" column, which corresponds to the sum of the individual context score values and thus is a simulated measure of combined targeting by all upregulated miRNAs. The column "Min. Aggregate PCT" indicates the minimal PCT value achieved by any of the miRNAs predicted to target a given transcript.
(4.13 MB XLS)

Table S7 MiRNAs that were ≥50% up- or down-regulated in Fetal hearts.
(0.03 MB XLS)

Figure S1 Identical miRNA expression in both control groups. Expression of miRNAs (ΔCT) in Sham (n = 6) versus Native (n = 7) hearts. Fitting represents linear regression ±95% confidence interval, n = 72, r^2^ = 0.99, slope = 1.00±0.01. Plotted and fitted are the expression data of all miRNAs with implications for the cardiovascular system (Table 1).
(0.07 MB TIF)

Figure S2 MiRNA expression in mechanical load-induced cardiac hypertrophy and cardiac atrophy. Relative expression data of all miRNAs that displayed a≥50% down-regulation in the AS group.
(4.44 MB TIF)
