# Correction: Common MicroRNA Signatures in Cardiac Hypertrophic and Atrophic Remodeling Induced by Changes in Hemodynamic Load

**DOI:** 10.1371/annotation/45ab2d79-0ce9-4832-a0d1-5274b5a1ddaa

**Published:** 2011-07-19

**Authors:** Ali El-Armouche, Alexander Peter Schwoerer, Christiane Neuber, Julius Emmons, Daniel Biermann, Thomas Christalla, Adam Grundhoff, Thomas Eschenhagen, Wolfram Hubertus Zimmermann, Heimo Ehmke

The 4th author's name is misspelled. The correct author name is: Julius Emons.

The corrected citation is: l-Armouche A, Schwoerer AP, Neuber C, Emons J, Biermann D, et al. (2010) Common MicroRNA Signatures in Cardiac Hypertrophic and Atrophic Remodeling Induced by Changes in Hemodynamic Load. PLoS ONE 5(12): e14263. doi:10.1371/journal.pone.0014263

